# A high-protein total diet replacement alters the regulation of food intake and energy homeostasis in healthy, normal-weight adults

**DOI:** 10.1007/s00394-021-02747-1

**Published:** 2021-12-20

**Authors:** Camila L. P. Oliveira, Normand G. Boulé, Sarah A. Elliott, Arya M. Sharma, Mario Siervo, Aloys Berg, Sunita Ghosh, Carla M. Prado

**Affiliations:** 1 Human Nutrition Research Unit, Department of Agricultural, Food and Nutritional Science, University of Alberta, Edmonton, AB Canada; 2 Alberta Diabetes Institute, University of Alberta, Edmonton, AB Canada; 3 Faculty of Kinesiology, Sport, and Recreation, University of Alberta, Edmonton, AB Canada; 4 Alberta Research Centre for Health Evidence, University of Alberta, Edmonton, AB Canada; 5 Division of Endocrinology and Metabolism, Department of Medicine, University of Alberta, Edmonton, AB Canada; 6 School of Life Sciences, Division of Physiology, Pharmacology and Neuroscience, University of Nottingham, Nottingham, England, UK; 7 Faculty of Medicine, University of Freiburg, Freiburg, Germany; 8 Department of Medical Oncology, University of Alberta, Edmonton, AB Canada

**Keywords:** Total diet replacement, Protein, Appetite, Appetite-related hormones, Energy homeostasis

## Abstract

**Purpose:**

Dietary intake can affect energy homeostasis and influence body weight control. The aim of this study was to compare the impact of high-protein total diet replacement (HP-TDR) versus a control (CON) diet in the regulation of food intake and energy homeostasis in healthy, normal-weight adults.

**Methods:**

In this acute randomized controlled, cross-over study, participants completed two isocaloric arms: a) HP-TDR: 35% carbohydrate, 40% protein, and 25% fat; b) CON: 55% carbohydrate, 15% protein, and 30% fat. The diets were provided for 32 h while inside a whole-body calorimetry unit. Appetite sensations, appetite-related hormones, and energy metabolism were assessed.

**Results:**

Forty-three healthy, normal-weight adults (19 females) participated. Appetite sensations did not differ between diets (all *p* > 0.05). Compared to the CON diet, the change in fasting blood markers during the HP-TDR intervention was smaller for peptide tyrosine-tyrosine (PYY; − 18.9 ± 7.9 pg/mL, *p* = 0.02) and greater for leptin (1859 ± 652 pg/mL, *p* = 0.007). Moreover, postprandial levels of glucagon-like peptide 1 (1.62 ± 0.36 pM, *p* < 0.001) and PYY (31.37 ± 8.05 pg/mL, *p* < 0.001) were higher in the HP-TDR. Significant correlations were observed between energy balance and satiety (*r* = − 0.41, *p* = 0.007), and energy balance and PFC (*r* = 0.33, *p* = 0.033) in the HP-TDR.

**Conclusion:**

Compared to the CON diet, the HP-TDR increased blood levels of anorexigenic hormones. Moreover, females and males responded differently to the intervention in terms of appetite sensations and appetite-related hormones.

**Trial registration:**

NCT02811276 (retrospectively registered on 16 June 2016) and NCT03565510 (retrospectively registered on 11 June 2018).

## Introduction

Food intake and energy homeostasis are key determinants of body weight control and regulated by several external and internal factors, such as the environment, individual’s physiology, and genetics [1]. Dysregulation in one or more of these factors can contribute to the development of obesity [2]. More specifically, the interplay between appetite-related hormones and the central nervous system has recently received special attention [3]. It has been demonstrated that individuals with obesity present with a decreased response or resistance to peripheral and central regulators of food intake and energy homeostasis [4]. In fact, an attenuated fall in postprandial ghrelin levels, leptin resistance, lower levels of peptide tyrosine-tyrosine (PYY), and glucagon-like peptide 1 (GLP-1) are usually present in obesity, as reviewed by Miller, Ullrey [5] and Perry, Wang [6]. These abnormal hormonal responses can affect food intake and energy homeostasis, potentially contributing to a state of positive energy imbalance (i.e., energy intake > energy expenditure [EE]). Therefore, strategies able to normalize these responses can ultimately help with the prevention and treatment of obesity. Importantly, food intake regulation differs between sexes, which can be partly explained by levels of gonadal steroid hormones [7] and neuronal responses to food intake [8]. For instance, estrogen exerts important effects on appetite sensations and appetite-related hormones, and has been shown to inhibit food intake and stimulate GLP-1 and leptin secretion [9, 10]. Additionally, females have a more intense satiety response compared to males, who are more inclined to overeat during ad libitum feeding [8]. Understanding the sex-based differences in food intake regulation is crucial when implementing nutritional interventions for the prevention and treatment of obesity.

Total diet replacements (TDR) and high-protein (HP) diets are two very popular weight management strategies [11]. Total diet replacements are nutritionally complete formula foods designed to replace the whole diet for a specific period of time to facilitate weight control. Nutritional strategies composed of these products have been shown to induce a significant and sustained body weight reduction in individuals with obesity [12–19]. High-protein diets are characterized by a protein content above recommended values (i.e., for healthy adults aged > 19 y: 0.80 g/kg of body weight/d or 10–35% of total energy intake) [20, 21] and have been demonstrated to facilitate weight management by increasing EE and satiety, and improving body composition [22]. Although the effects of these dietary strategies in isolation on components related to food intake and energy homeostasis have been investigated [23, 24], the combined effects of a high-protein total diet replacement (HP-TDR) have not yet been explored, despite their worldwide availability and high consumption by individuals with normal or excess body weight.

Therefore, the assessment of the effects of a HP-TDR on components related to food intake and energy homeostasis is crucial. This is especially important in the context of a controlled environment using a state-of-the-art methodology in healthy individuals with a normal body weight. Such study design characteristics eliminate confounding effects of obesity and comorbidities, allowing a better understanding of the impact of this strategy in a normal physiological condition. This will provide further insight into the potential role of a HP-TDR for weight maintenance and prevention of overweight and obesity. Additionally, findings from this study can then be used as reference for studies testing the effects of HP-TDR in individuals with overweight and obesity.

Considering the aspects discussed above, the aim of this inpatient metabolic balance study was to compare the acute impact of HP-TDR versus a control (CON) diet (North American) on appetite sensations, appetite-related hormones and its association with energy metabolism components in healthy, normal-weight adults of both sexes. We hypothesized that, compared to the CON diet, participants consuming the HP-TDR would feel more satiated, have higher blood levels of anorexigenic hormones (i.e., GLP-1, PYY, and leptin), lower levels of the orexigenic hormone ghrelin, and that appetite sensations and appetite-related hormones would correlate with energy metabolism components. Additionally, we hypothesized that females would experience increased satiety and increased blood levels of leptin during the HP-TDR and CON interventions, and that there would be no other differences in appetite sensations and appetite-related hormones between sexes.

## Methods

### Study design and participants

 This was a planned secondary analysis of a previously described randomized, controlled, crossover inpatient study conducted separately in females and males; full methodological details and findings on changes in energy metabolism have been published [25]. Briefly, participants were invited to participate in this study by poster advertisements placed on notice boards at the University of Alberta north campus. Healthy adults aged 18–35 years were recruited, with a body mass index (BMI) ranging from 18.5 to 24.9 kg/m^2^. Females were required to have a regular menstrual cycle. Potential participants were excluded if they had any diagnosed disease, were smokers, were taking any medication and/or nutritional supplement that could affect energy metabolism and/or body composition, had any dietary restrictions, were performing > 1 h/day or > 7 h/week of exercise, were recently exposed to tests involving radiation, had claustrophobia, and if females were pregnant or lactating.

### Experimental protocol

**Fig. 1:**
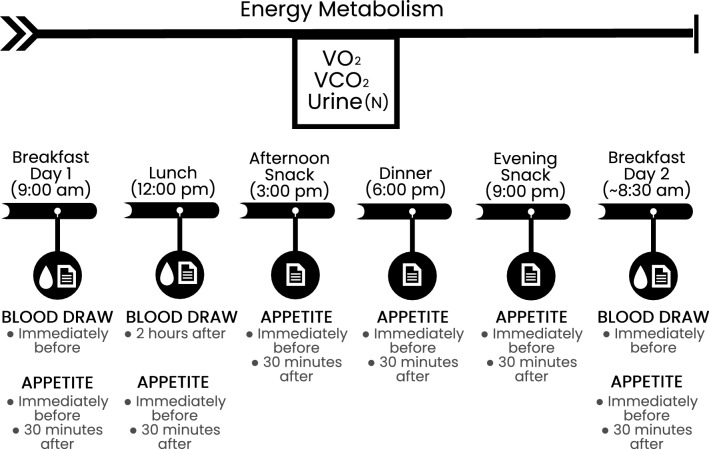
Overview of the experimental protocol. *VO*_*2*_ volume of oxygen, *VCO*_*2*_ volume of carbon dioxide, *N* nitrogen

Eligible participants were enrolled in the study and randomly assigned to begin with a HP-TDR or CON diet. Participants consumed these isocaloric diets on separate intervention days for 32 consecutive hours while inside a whole-body calorimetry unit (WBCU) for the measurement of energy metabolism components, appetite sensations, and appetite-related hormones (Fig. 1). Females were required to be in the follicular phase of their menstrual cycle during each 32-h intervention phase. A wash-out period of approximately 1 month was required for females and 2 weeks for males. A 3-day run-in period with a controlled, energy-balanced diet preceded both intervention phases. At baseline, participant’s height, weight, waist circumference, body composition (GE Lunar iDXA, General Electric Company, Madison, USA; enCORE software 13.60 Lunar iDXA GE Health Care®) and resting metabolic rate (RMR) were assessed.

### Diets

Immediately before each 32-h intervention period in the WBCU, participants received an eucaloric diet for three consecutive days and were instructed not to eat any other food item, except for calorie-free beverages, and not to consume any caffeinated food product. Moreover, participants were asked to maintain their physical activity levels as low as possible for the first two days and not to exercise on the third day. They received a breakfast, lunch, dinner, and two snacks per day and the macronutrient distribution was similar to the CON intervention (i.e., 55% carbohydrate, 15% protein, and 30% fat), which resembled the North American dietary pattern [26, 27]. The energy content of the diet was calculated to maintain participants in energy balance and was based on the RMR test performed at baseline multiplied by a physical activity factor [20] and a coefficient representing the thermic effect of food (i.e., 1.075) [28], fully described elsewhere [25].

Following this 3-day run-in period, participants entered the WBCU and stayed for 32 consecutive hours while consuming a HP-TDR and a CON diet in a random order. The first dietary intervention was designed to maintain participants in energy balance and the second matched its energy content (i.e., isocaloric). The energy contents of the meals and snacks were also matched for the HP-TDR and CON diets. During each intervention period, a breakfast (9:00 am), lunch (12:00 pm), dinner (6:00 pm), and two snacks (3:00 pm and 9:00 pm) were provided on day 1 and a breakfast (~ 8:30 am) on day 2. Additionally, bottled water was provided ad libitum. The HP-TDR consisted of a soy-protein nutritional supplement (Almased®, Almased USA, Inc., Wellington, FL, USA) mixed with olive oil and low-fat milk (1% fat) (for the main meals) or apple juice (for the snacks), per label instructions [29]. The CON diet was composed of regular food items (i.e., breakfast: bread, peanut butter, and orange juice; lunch: turkey wrap and tomato soup; afternoon snack: apple, crackers, and cheese; dinner: chicken stir fry and brown rice; evening snack: cereal, milk, and almonds), fully described elsewhere [25]. Participants were required to consume all the food provided and meal trays were checked after consumption. The nutrient content of the dietary interventions is described in Table 1. The run-in and the intervention diets were designed by a registered dietitian using the Food Processor Nutrition Analysis Software (version 11.0.124, ESHA Research, Salem, OR, USA) and prepared at the metabolic kitchen of the Human Nutrition Research Unit (HNRU).

**Table 1:** Nutrient content of the intervention diets

	HP-TDR			CON		
	Females	Males	Sex difference^a^	Females	Males	Sex difference^a^
Energy						
kcal/day	2008 ± 167	2225 ± 250	0.002	2007 ± 167	2224 ± 250	0.002
Kcal/kg/day	33.0 ± 2.5	33.4 ± 3.9	0.67	32.9 ± 2.5	33.4 ± 3.9	0.67
Protein						
% energy	39.9 ± 0.2	39.8 ± 0.3	0.35	15.4 ± 0.3	15.2 ± 0.2	0.02
g/day	199 ± 16	220 ± 24	0.002	78 ± 6	86 ± 9	0.007
g/kg/day	3.3 ± 0.2	3.3 ± 0.4	0.72	1.3 ± 0.1	1.3 ± 0.1	0.978
Fat						
% energy	25.0 ± 0.3	24.9 ± 0.1	0.85	30.3 ± 0.5	30.2 ± 0.0	0.29
g/day	55 ± 4	61 ± 6	0.004	68 ± 5	76 ± 8	0.001
g/kg/day	0.9 ± 0.7	0.9 ± 0.1	0.71	1.1 ± 0.1	1.1 ± 0.1	0.72
Carbohydrate						
% energy	35.1 ± 0.3	35.2 ± 0.3	0.30	54.2 ± 0.6	54.6 ± 0.2	0.04
g/day	175 ± 14	195 ± 22	0.001	277 ± 24	309 ± 34	0.001
g/kg/day	2.9 ± 0.2	2.9 ± 0.3	0.62	4.5 ± 0.4	4.6 ± 0.5	0.62
Sugars (g/day)	169 ± 13	188 ± 22	0.002	86 ± 9	97 ± 12	0.002
Fiber (g/day)	4 ± 0	4 ± 0	0.003	27 ± 2	31 ± 3	< 0.001
Saturated fat (g/day)	11 ± 1	12 ± 1	0.001	16 ± 2	17 ± 2	0.23
Monounsaturated fat (g/day)	34 ± 2	37 ± 3	0.003	28 ± 3	33 ± 3	< 0.001
Polyunsaturated fat (g/day)	5 ± 0	5 ± 0	0.004	16 ± 1	17 ± 1	0.001
Cholesterol (mg/day)	34 ± 10	42 ± 7	0.005	103 ± 55	110 ± 16	0.007

Data are expressed as mean ± standard deviation

*n* = 43 (females: *n* = 19; males: *n* = 24)

A version of this table has been published elsewhere [27]

*SD* standard deviation, *HP-TDR* high-protein total diet replacement, *CON* control

^a^*P* values represent the difference between females and males and were detected with the use of an independent-samples *t *test or a Mann–Whitney *U* test, as appropriate

### Energy metabolism

During each dietary intervention period, participants stayed for 32 consecutive hours (8:00 am on day 1 until 4:00 pm on day 2) inside an open-circuit WBCU, where the volume of oxygen (VO_2_) and carbon dioxide (VCO_2_) were continuously measured. The whole-body calorimetry conditions included a strict and standard schedule fully described elsewhere [25]. In short, participants were asked to keep a minimum physical activity level throughout the 32-h tests, a 40-min walking exercise session was performed on the morning of the first day of the test (10:20 am) on a treadmill (BH Fitness T8 SPORT, BH Fitness, Foothill Ranch, Calif., USA), and sleep was only allowed during the night. Additionally, participants were instructed to collect their urine the entire time for nitrogen excretion analysis. Using the formula of Brouwer [30], EE, macronutrient oxidation rates and balances were calculated, a method fully described elsewhere [25].

### Appetite sensations

During the HP-TDR and CON interventions, appetite sensations were rated (i.e., hunger, satiety, fullness, and prospective food consumption [PFC]) immediately before and 30 min after each meal and snack using a validated anchored 100-mm visual analog scale (VAS) [31]. A total of 12 assessments were performed throughout each intervention period using a paper-and-pen method and data have been double-entered to ensure quality. Questions were worded as follows: “How hungry do you feel?”, “How satisfied do you feel?”, “How full do you feel?”, and “How much do you think you can eat?” Answers for each of those questions were anchored as: “I am not hungry at all” to “I have never been more hungry”, “I am completely empty” to “I cannot eat another bite”, “Not at all full” to “Totally full”, and “Nothing at all” to “A lot”, respectively. Appetite sensation responses were evaluated by calculating the 24-h area under the curve (AUC) with the trapezoid method [32]. Moreover, the composite satiety score (CSS) was calculated at each time of measurement using the following equation: CSS (mm) = (satiety + fullness + (100 − PFC) + (100 − hunger))/4 [33]. A higher CSS is associated with a higher satiety sensation and a subsequent lower motivation to eat.

### Blood analyses

Blood was sampled by venipuncture at three time points during each intervention period 1) the morning on the first day of test (fasting day 1, 7:30 am); 2) two hours after lunch (postprandial, 2:30 pm); and 3) the morning on the second day of test (fasting day 2, 8:00 am). These time points were chosen to compare differences in blood markers in fasting and postprandial states. Both morning blood draws were sampled from participants after a 10- to 12-h overnight fast using BD Vacutainer® blood collection tubes (Becton, Dickinson and Company, Franklin Lakes, NJ, USA), spray-coated with silica and a polymer gel for serum separation or with K2-ethylenediaminetetraacetic acid (EDTA) for plasma separation. Serum samples were analyzed for leptin and plasma samples were analyzed for ghrelin (active), PYY, and GLP-1 (active). Before centrifugation, a protease inhibitor 4-(2-aminoethyl) benzenesulfonyl fluoride hydrochloride (AEBSF) (Sigma-Aldrich, Oakville, ON, Canada) was added to the K2-EDTA tubes and after it, hydrochloric acid (1 N,100 μL) was added to the ghrelin aliquot. Leptin and GLP-1 were measured by electro-chemiluminescence using the MULTI-ARRAY® Assay System (Meso Scale Discovery®, Gaithersburg, MD, USA) and V-PLEX® (Meso Scale Discovery®, Gaithersburg, MD, USA), respectively. Ghrelin and PYY were measured by enzyme-linked immunosorbent assay kits from EMD Millipore Co. (Billerica, MA, USA). All analyses were performed at the HNRU according to manufacturer’s instructions, in duplicates, and were repeated when they had not fallen within the range of the standard curves. The coefficients of variation (CV) were 3.67% and 6.99% for leptin, 6.10% and 5.82% for ghrelin, 7.48% and 10.3% for PYY, and 5.34% and 5.24% for GLP-1 in females and males, respectively.

### Statistical analysis

Sample size calculation for the primary study has been described elsewhere [25]. An additional sample size calculation was conducted for this planned, secondary analysis based on differences in satiety between dependent groups receiving a HP diet (973 ± 178 mm/24 h) or an adequate protein diet (765 ± 304 mm/24 h) from a previously published study [23]. Group sample sizes of 14 in each arm would achieve 80% power to detect a difference of 208 mm/24 h with a two-sided significance level of 0.05. Assuming a 20% attrition rate, a total 17 participants would be needed in each group. The sample size calculation was done using a software developed by David Schoenfeld (http://hedwig.mgh.harvard.edu/sample_size/js/js_crossover_quant.html). The study was not powered to assess differences by sex in appetite sensations and appetite-related hormones. For this reason, no further power calculations were performed. Analyzing the data by sex was for the purpose of descriptive statistics only.

Data were expressed as mean ± standard deviation (SD) for continuous variables and mean ± standard error of the mean (SEM) to report differences between sexes and groups. Shapiro–Wilk test was used to check the normality of the data. Independent samples *t *test or Mann–Whitney *U* tests were used as appropriate to compare nutrient intake and baseline appetite-related hormones between sexes. Possible differences between the HP-TDR and CON diets were explored using a one-way repeated measures analysis of variance (ANOVA). Mixed ANOVA with within-subject factors (i.e., dietary interventions and/or time) and a between-subjects factor (i.e., sex) was used to analyze the data by sex. Post hoc analyses were applied with all ANOVA tests using a Tukey’s test (equal variances assumed) or Games-Howell (equal variances not assumed). Diagnostics, such as the assessment of normality, homogeneity of variances using the Box’s test of equality of covariance matrices and Levene’s test for equality of variances were used to check if the ANOVA assumptions were valid. Partial correlation analyses controlling for sex were performed between energy metabolism components, appetite sensations, and appetite-related hormones. IBM® SPSS® Statistics version 24 (International Business Machines Corporation, New York, NJ, USA) was used to perform all statistical analyses. A *p* < 0.05 was used for most of the comparisons and test specific *p* values were used for multiple comparisons.

## Results

### Participants

A total of 43 healthy adults (*n* = 19 females and *n* = 24 males) completed the study and were included in the analyses. Characteristics of study participants are shown in Table 2, with a more extensive description presented previously [27]. Briefly, they were primarily a group of white healthy adults in their 20 s, with a normal BMI, and active. Most of them (79%) were not taking any medication or nutritional supplement, 12% were taking multivitamin/mineral, 7% antidepressant (stable dose for previous 3 months), and 2% antihistamine. Compared to males, females were shorter, had lower body weight, smaller waist circumference, higher fat mass (FM), lower lean soft tissue (LST) and bone mineral content (BMC), all *p* < 0.05. There was no difference in the volume of liquid consumed during the CON and HP-TDR diets (*p* = 0.651, data not shown).

**Table 2:** Baseline characteristics of the study participants

Characteristics	Females	Males
Age (years)	25 ± 3	23 ± 4
Height (cm)	166.3 ± 5.7	174.9 ± 6.1
Weight (kg)	61.1 ± 4.8	67.0 ± 7.3
Waist circumference (cm)	71.4 ± 2.8	76.9 ± 6.1
BMI (kg/m^2^)	22.2 ± 1.2	21.9 ± 1.6
FM (kg)	18.6 ± 3.3	12.7 ± 4.9
LST (kg)	40.1 ± 4.4	51.4 ± 5.6
BMC (kg)	2.4 ± 0.2	2.9 ± 0.3
Race		
White	7 (37)	12 (50)
Asian	5 (26)	9 (37)
Hispanic	3 (16)	0 (0)
Black	1 (5)	0 (0)
Other	3 (16)	3 (13)
Physical activity level^a^		
Insufficiently active	1 (6)	1 (4)
Moderately active	5 (26)	2 (8)
Active	13 (68)	21 (88)

Data are expressed as mean ± standard deviation or *n* (%)

*n* = 43 (females: *n* = 19; males: *n* = 24)

A version of this table has been published elsewhere [27]

*SD* standard deviation, *BMI* body mass index, *FM* fat mass, *LST* lean soft tissue, *BMC* bone mineral content

^a^Physical activity levels were classified according to the Godin–Shephard Leisure­Time Physical Activity Questionnaire

### Energy metabolism

**Fig. 2:**
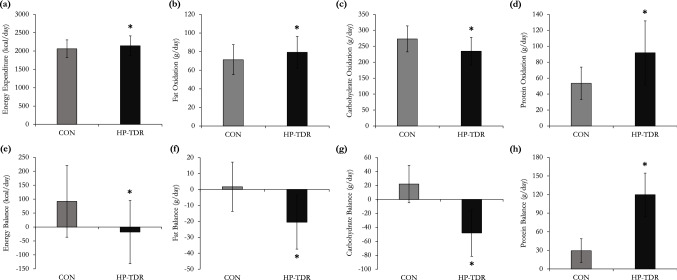
Energy expenditure (panel **a**), macronutrient oxidation rates (panels **b**, **c**, and **d**), energy balance (panel **e**), and macronutrient balances (panels **f**, **g**, and **h**) during the CON and HP-TDR interventions. Values are mean (standard deviation). *N* = 43 (females *N* = 19; males *N* = 24). *Significant difference between the HP-TDR and CON diets, *p* < 0.01 as assessed by a mixed analysis of variance. *CON* control, *HP-TDR* high-protein total diet replacement. This data have been reported in detail elsewhere [27]

Energy metabolism components assessed during the HP-TDR and CON diets are illustrated in Fig. 2 and reported in detail elsewhere [27]. While consuming the HP-TDR, participants presented higher 24-h EE (*p* < 0.001), protein and fat oxidation rates (*p* = 0.013 and *p* < 0.001, respectively), and lower carbohydrate oxidation rate (*p* < 0.001). Additionally, the HP-TDR led to greater negative energy (*p* < 0.001), fat (*p* < 0.001), and carbohydrate (*p* < 0.001) imbalance, and a greater positive imbalance for protein (*p* < 0.001), compared to the CON diet. The order in which participants received the dietary interventions did not affect any of the energy metabolism variables analyzed (all *p* > 0.05).

### Appetite sensations

**Fig. 3:**
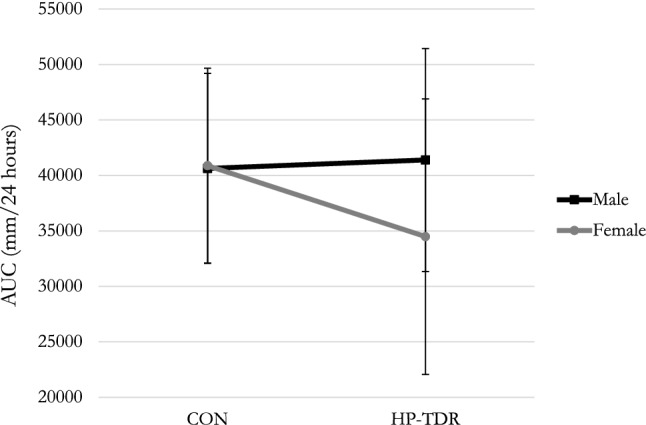
Interaction between diet and sex on 24-h AUC for PFC (*p* = 0.04). Data are presented as mean ± standard deviation. *N* = 43 (*N* = 19 females; *N* = 24 males). In females, the 24-h AUC for PFC was lower with the HP-TDR compared to the CON diet, *p* = 0.04 as assessed by a post hoc test for a mixed analysis of variance. In the HP-TDR diet, the 24-h AUC for PFC was lower in females compared to males, *p* = 0.05 as assessed by a post hoc test for a mixed analysis of variance. *AUC* area under the curve, *CON* control, *HP-TDR* high-protein total diet replacement, *PFC* prospective food consumption

The 24-h AUC for hunger, satiety, fullness, and PFC did not differ between diets (all *p* > 0.05). There was a significant interaction between diet and sex on 24-h AUC for PFC (*p* = 0.04), Fig. 3. In the HP-TDR diet, the 24-h AUC for PFC was lower in females compared to males (− 6908 ± 3424 mm/24 h; *p* = 0.05), while no significant difference between sexes was observed in the CON diet (253 ± 2659 mm/24 h; *p* = 0.92). In females, the 24-h AUC for PFC was lower with the HP-TDR compared to the CON diet (− 6399 ± 2940 mm/24 h; *p* = 0.04), but no significant difference between the diets was observed in males (761 ± 2101 mm/24 h; *p* = 0.72). Significant correlations were noted between energy balance and satiety, as well as between energy balance and PFC only when participants were fed the HP-TDR, Table 3.There was no significant interaction between diet, sex, and time (*p* = 0.45). The order in which participants received the dietary interventions did not affect any of the appetite sensations analyzed (all *p* > 0.05).

**Table 3:** Partial correlation analyses (controlling for sex) between energy metabolism components and 24-h area under the curve for each appetite sensation

		Hunger	Satiety	Fullness	PFC
Energy expenditure (kcal/day)	HP-TDR	− 0.07	0.12	0.04	0.01
	CON	0.06	− 0.11	− 0.06	0.07
Fat oxidation (g/day)	HP-TDR	− 0.21	0.23	0.1	− 0.16
	CON	0.04	0.10	0.09	− 0.02
Protein oxidation (g/day)	HP-TDR	0.10	− 0.15	− 0.16	0.05
	CON	− 0.12	0.08	0.08	− 0.30
Carbohydrate oxidation (g/day)	HP-TDR	0.03	0.06	0.09	0.12
	CON	0.09	− 0.25	− 0.19	0.22
Energy balance (kcal/day)	HP-TDR	0.28	− 0.41^a^	− 0.25	0.33^b^
	CON	− 0.03	0.16	0.15	− 0.10
Fat balance (g/day)	HP-TDR	0.24	− 0.26	− 0.13	0.22
	CON	− 0.02	− 0.11	− 0.09	0.03
Protein balance (g/day)	HP-TDR	− 0.06	0.11	0.11	0.06
	CON	0.10	− 0.04	− 0.02	0.25
Carbohydrate balance (g/day)	HP-TDR	0.01	− 0.12	− 0.16	− 0.05
	CON	− 0.05	0.32^b^	0.27	− 0.27

*n* = 43 (females *n* = 19; males *n* = 24)

*HP-TDR* high-protein total diet replacement, *CON* control, *PFC* prospective food consumption

^a^*p* < 0.01

^b^*p* < 0.05

### Appetite-related hormones

Appetite-related hormones assessed in a fasting state on days 1 and 2, and after lunch during the HP-TDR and CON diets are shown in Table 4. Due to difficult draw or blood markers falling below the limit of detection, the sample size was not the same for all appetite-related hormones. Before the dietary interventions (i.e., fasting day 1), blood levels of GLP-1, PYY, and leptin were different between sexes. Compared to males, females presented lower levels of GLP-1 (HP-TDR: − 2.15 ± 0.82 pM, *p* = 0.015; CON: − 2.22 ± 0.96 pM, *p* = 0.02) and PYY (HP-TDR: − 85.7 ± 13.9 pg/mL, *p* < 0.001; CON: − 94.5 ± 12.6 pg/mL, *p* < 0.001), and higher levels of leptin (HP-TDR: 17,068 ± 1594 pg/mL, *p* < 0.001; CON: 18,562 ± 2134 pg/mL, *p* < 0.001).

**Table 4:** Appetite-related hormones during the HP-TDR and CON diets

	HP-TDR			CON			∆^a^		Postprandial^b^	
	Fasting Day 1	Postprandial	Fasting Day 2	Fasting Day 1	Postprandial	Fasting Day 2	Diet *x* Sex	Diet effect	Diet *x *Sex	Diet effect
Ghrelin (pg/mL)^c^	442.76 ± 296.12	374.96 ± 262.06	441.91 ± 251.69	470.97 ± 313.84	365.90 ± 222.92	424.88 ± 258.08	0.96	0.07	0.04	0.67
GLP-1 (pM)^d^	1.62 ± 3.32	4.21 ± 5.19	1.15 ± 3.09	1.62 ± 3.28	2.59 ± 4.18	1.48 ± 3.26	0.39	0.05	0.003	< 0.001
PYY (pg/mL)^e^	124.0 ± 62.3	163.4 ± 75.1	97.9 ± 51.5	123.4 ± 62.7	132.0 ± 56.5	114.9 ± 56.4	0.57	0.02	0.03	< 0.001
Leptin (pg/mL)^c^	8702 ± 9994	7422 ± 9075	12,591 ± 14,951	9522 ± 11,155	8367 ± 10,247	11,552 ± 13,591	0.006	0.007	0.25	0.24

Data are expressed as mean ± standard deviation

*HP-TDR* high-protein total diet replacement, *CON* control, *GLP-1* glucagon-like peptide 1, *PYY* peptide tyrosine–tyrosine

^a^*P* values represent the effect of the interventions on the change from fasting day 1 to fasting day 2 and were detected with the use of a mixed analysis of variance

^b^*P* values represent the effect of the interventions on postprandial values and were detected with the use of a mixed analysis of variance

^c^∆: *n* = 43 (*n* = 19 females, *n* = 24 males); Postprandial: *n* = 42 (*n* = 18 females, *n* = 24 males)

^d^∆: *n* = 38 (*n* = 14 females, *n* = 24 males); Postprandial: *n* = 37 (*n* = 13 females, *n* = 24 males)

^e^∆: *n* = 40 (*n* = 16 females, *n* = 24 males); Postprandial: *n* = 42 (*n* = 18 females, *n* = 24 males)

Compared to the CON diet, the change in fasting blood markers with the HP-TDR was smaller for PYY (− 18.9 ± 7.9 pg/mL, *p* = 0.02) and greater for leptin (1859 ± 652 pg/mL, *p* = 0.007). There was a significant interaction between diet and sex on the change from fasting day 1 to fasting day 2 in leptin concentration (*p* = 0.006,). In both sexes, this change was higher with the HP-TDR intervention (females: 3803 ± 1361 pg/mL, *p* = 0.01; males: 320 ± 113 pg/mL, *p* = 0.01). In both intervention groups, the change in this biomarker was greater in females compared to males (HP-TDR: 8042 ± 1214 pg/mL, *p* < 0.001; CON: 4559 ± 1140 pg/mL, *p* < 0.001).

Postprandial levels of GLP-1 (1.62 ± 0.36 pM, *p* < 0.001) and PYY (31.3 ± 8.0 pg/mL, *p* < 0.001) were higher in the HP-TDR compared to the CON diet. There was a significant interaction between diet and sex on ghrelin (*p* = 0.048), GLP-1 (*p* = 0.003), and PYY (*p* = 0.03). In the HP-TDR diet, postprandial ghrelin concentration was higher in females compared to males (197.33 ± 74.36 pg/mL, *p* = 0.01). Postprandial GLP-1 (HP-TDR: − 5.35 ± 1.46 pM, *p* = 0.001; CON: − 3.19 ± 1.22 pM, *p* = 0.01) and PYY (HP-TDR: − 97.1 ± 17.9 pg/mL, *p* < 0.001; CON: − 60.7 ± 15.0 pg/mL, *p* < 0.001) were lower in females compared to males in both diets. In males, postprandial levels of GLP-1 and PYY were higher in the HP-TDR compared to the CON diet (GLP-1: 2.38 ± 0.49 pM, *p* < 0.001; PYY: 46.0 ± 7.4 pg/mL, *p* < 0.001), while in females, these markers were not different between dietary interventions (GLP-1: 0.21 ± 0.12 pM, *p* = 0.10; PYY: 11.7 ± 15.0 pg/mL, *p* = 0.44). The order in which participants received the dietary interventions did not affect any of the appetite-related hormones analyzed (all *p* > 0.05).

## Discussion

The primary findings of our study were that compared to a standard North American diet (CON), the HP-TDR increased blood levels of anorexigenic hormones and reduced the PFC in females. Moreover, correlations were observed between energy balance and satiety, as well as between energy balance and PFC. Interestingly, females and males responded differently to the dietary intervention. While females presented with a response to the HP-TDR in terms of appetite sensations, males’ response to this intervention was mostly on appetite-related hormones. These results highlight the impact a HP-TDR consumption has on appetite sensations, appetite-related hormones, and energy metabolism components of healthy, normal-weight adults and provides further insight into the impact of this strategy in the regulation of food intake and energy homeostasis. Additionally, the different findings between males and females may potentially impact the prescription of future nutritional weight management strategies that are sex-specific.

In our study, only females experienced a decrease in appetite with the HP-TDR characterized by a reduced 24-h AUC for PFC. These results suggest that female’s appetite sensations are more sensitive and reactive to dietary manipulation than male’s, which is in line with previous nutritional intervention studies [8, 34–36]. In a similar design, Westerterp-Plantenga et al. [35] observed a more pronounced decrease in hunger and increase in satiety in females in response to an acute dietary intervention composed of 30% of protein. The differences observed in appetite sensations between sexes can be partly explained by the gonadal steroid hormones [7] and neuronal responses to food intake [8]. Gonadal steroid hormones are able to influence neural processing of peripheral feedback signals that control eating, such as ghrelin, cholecystokinin, glucagon, insulin, and leptin [7]. More specifically estrogen seems to have an inhibitory effect on food intake, which is reflected during the follicular phase of the menstrual cycle when this hormone’s level is relatively high and female’s energy intake is lower than the other phases of the cycle [9, 37]. In fact, in our study, females were evaluated during the follicular phase, which could have impacted their response to the dietary intervention, accentuating the decrease in appetite. For this reason, further research is needed to examine if the decrease in appetite persists during other phases of the menstrual cycle with this nutritional intervention. Notwithstanding the influences of the follicular phase, a higher protein intake has been demonstrated to improve satiety, being the most satiating macronutrient, followed by carbohydrate, and fat [38]. Therefore, irrespective of the menstrual cycle, a higher protein intake is likely the biggest contributor to the decrease in appetite observed in our study. In addition to the macronutrient composition of the intervention diets, the physical state of the food has been shown to impact appetite sensations [39]. In fact, Martens et al. [40] showed that a high-protein solid meal evoked stronger suppression of hunger and desire to eat than a liquid meal of identical nutrient profile in healthy, normal-weight, young adults. Considering that in this study females presented with a reduced PFC with a liquid diet, it is possible that other factors such as the diet’s macronutrient composition might have had a stronger effect on appetite suppression than its physical state.

Although males did not experience a direct change in appetite sensations with the dietary intervention, a partial correlation analysis controlling for sex revealed a negative correlation between energy balance and satiety, and a positive correlation between energy balance and PFC only in the HP-TDR arm. These findings are very interesting, considering that participants were in negative energy imbalance during the HP-TDR intervention. A state of negative energy imbalance has been shown to provoke a compensation in energy intake in the short term and a metabolic adaptation over the long term, contributing to the onset and progression of obesity [41]. This compensation is partly driven by an increase in hunger [42]. Therefore, strategies able to control one or more factors leading to an energy compensation in response to a state of negative energy imbalance are crucial in the prevention and treatment of obesity. Interestingly, even though participants were in a state of positive energy balance with the CON intervention, no significant correlations were found between energy balance and appetite sensations in this group. Hence, results from our study suggest that an HP-TDR might be an interesting strategy to counteract the increased hunger and, consequently, an energy compensation in the presence of a state of negative energy imbalance.

Regarding the change from fasting day 1 to fasting day 2 in appetite-related hormones, our study showed an interaction between diet and sex in leptin levels. In both intervention groups, the change in this biomarker was greater in females. Additionally, in both sexes, this change was higher with the HP-TDR intervention. Leptin is a hormone produced primarily by white adipose tissue and its plasma levels are positively correlated with total body fat [2]. Although in this study females presented with higher FM than males, their leptin levels were higher at baseline (i.e., fasting day 1) even after controlling for FM (results not shown, *p* < 0.001). This difference at baseline partly explains the greater change observed in this biomarker in females, which is in line with previous studies [43, 44]. Additionally, estrogen seems to stimulate leptin secretion in healthy females [45]. Notably, the HP-TDR used contained soy isoflavones [46], a natural estrogen-like compound which may increase female’s estrogen levels. This could have accentuated sex-differences in leptin, further explaining the results presented herein. It has been demonstrated that leptin decreases food intake and increases EE by acting directly in the hypothalamus and controlling regions that regulate food intake and energy homeostasis [2, 3]. Although promising, increased leptin level does not lead to decreased food intake and increased EE in individuals presenting with obesity, which is explained by a state of leptin resistance [2]. Therefore, increasing leptin levels via medications and/or nutritional strategies is not an effective strategy for treating obesity, but it can be a promising strategy for preventing it. In our study, the change from fasting day 1 to fasting day 2 in leptin was higher with the HP-TDR intervention. Although we are unable to quantify the variation in food intake and EE resulted from this change, this contributes to the prevention of obesity; since it is a multifactorial disease, every contribution counts [47]. For instance, an intervention able to reduce energy intake by as little as 50 kcal/day could offset weight gain in 90% of the population [48]. Therefore, the observed increase in fasting leptin levels in this study is promising, considering the physiological effects of this hormone on the control of food intake and energy homeostasis in individuals not diagnosed with obesity.

Regardless of sex, our study showed an increase in postprandial levels of GLP-1 and PYY with the HP-TDR. Glucagon-like peptide 1 is considered a biomarker of satiation and is produced primarily in the ileum in response to the presence of nutrients [49]. Its main functions involve the stimulation of insulin release and adjustments of stomach and gut motility, which contribute to its role as an appetite-suppressing peptide [50]. Peptide tyrosine–tyrosine is another gut-derived peptide that is released from the colon and has been shown to suppress food intake in individuals with normal weight and obesity [50]. The production of both gut-derived peptides has been shown to increase with HP meals [23, 51, 52]. Considering the macronutrient composition of the HP-TDR, it is very likely that its protein content was the main contributor to the increased levels of GLP-1 and PYY observed in our study. Interestingly, only males experienced an increase in postprandial levels of GLP-1 and PYY, and a decrease in postprandial ghrelin with the HP-TDR intervention. Dietary protein has been shown to suppress postprandial ghrelin production [53] and, as mentioned above, increases GLP-1 and PYY [23, 51, 52], partially explaining the result presented herein. In addition to that, males presented with higher baseline blood levels of GLP-1 and PYY (i.e., fasting day 1), also contributing to the observed results. Although females did not experience a similar response with the HP-TDR, it is possible that the phase of the menstrual cycle could have contributed to the lack of effect observed. Estrogen and progesterone have important effects on appetite-related hormones [10] and, considering females were tested during the follicular phase of the menstrual cycle when these hormones are at their lowest concentration, it is possible that the stimuli were not enough to affect these gut-derived peptides.

This study has several strengths, including the well-controlled design, the dietary intervention, and the use of state-of-the-art techniques for the assessment of energy metabolism. Some potential limitations are the acute dietary intervention, the specificity of the population group (i.e., healthy, normal-weight young adults), a selective menstrual cycle phase, a limited number of appetite sensation assessments throughout each intervention period, the lack of assessment on diet liking, the standardized meal portions, the physical state of the HP-TDR (i.e., liquid), the single protein source of the HP-TDR (i.e., soy protein), and the fact that the carbohydrate and fat contents of the diets were not matched. In addition, because the CON and HP-TDR diets were eucaloric, the observed changes in appetite sensations and appetite-related hormones could not be directly translated into changes in energy intake. However, it is possible that under ad libitum conditions, the observed changes in biomarkers of appetite regulation may lead to changes in food intake over the long term, and subsequently changes in body weight and composition. Considering that the length of the washout period was different between females and males, it is possible that the memory effects of the interventions on participant’s behavior differed by sex. Moreover, some of the analyses might be underpowered as this is a secondary analysis of a previously described clinical trial [25]. These limitations restrict our ability to translate the results presented herein to longer intervention periods, other population groups, to other menstrual cycle phases, and to other types of HP-TDR. Therefore, future studies are needed to better understand the long-term effects of this type of intervention on the mechanisms involved in the regulation of food intake and energy homeostasis of healthy and diseased population groups in more than one menstrual cycle phase.

In conclusion, this study showed that compared to a standard North American diet, an isocaloric HP-TDR increased blood levels of anorexigenic hormones and reduced the PFC in females. Moreover, correlations were observed between energy balance and satiety, as well as between energy balance and PFC. Females’ response to the HP-TDR was related to appetite sensations, while males’ was mostly related to appetite hormones. These results provide further insight into the impact of this dietary strategy in the regulation of food intake and energy homeostasis.
